# Greater Occipital Nerve Block as an Opioid-Sparing Alternative in Scalp Surgery: A Case Series

**DOI:** 10.7759/cureus.84240

**Published:** 2025-05-16

**Authors:** Jorge Carteiro, Nuno Torres, Idalina Rodrigues, Lucindo Ormonde

**Affiliations:** 1 Anesthesiology, Unidade Local de Saúde (ULS) Santa Maria, Lisboa, PRT

**Keywords:** case report, greater occipital nerve, opioid-sparing analgesia, postoperative occipital pain, scalp surgery

## Abstract

The greater occipital nerve (GON) is primarily responsible for the sensory innervation of the posterior region of the scalp. GON block has been described as a safe and effective therapeutic option for various types of headaches, including occipital neuralgia, migraine, cervicogenic headache, and cluster headache. Despite its established role in headache management, there is a paucity of evidence in the literature regarding its use in the perioperative setting, particularly in surgeries involving the occipital region of the scalp.

In this context, we present a case series of three patients undergoing excision of malignant tumors in the occipital scalp region, followed by reconstruction. GON block was performed following anesthetic induction to evaluate its efficacy in postoperative pain control. The results suggest that this technique may provide effective analgesia while also contributing to reduced opioid consumption.

## Introduction

Peripheral nerve blocks are well-established in headache treatment, with a greater occipital nerve (GON) block being widely utilized and supported by evidence [1,2]. While the GON block may have applications beyond headache treatment, including in surgical procedures involving the occipital region of the scalp, the literature on its use in this perioperative context remains sparse [3,4].

Anatomically, the GON is the largest sensory nerve supplying the occipital scalp. It originates from the dorsal ramus of the second cervical vertebra (C2) spinal nerve, ascending through the fascial plane between the inferior oblique and semispinalis capitis muscles. The nerve fibers pierce the semispinalis capitis muscle and pass deep to the trapezius muscle, emerging through the aponeurosis just inferior to the superior nuchal line. At this point, it assumes a subcutaneous course and is typically accompanied by the occipital artery, which most often lies lateral to the GON [5,6].

Nociceptive stimuli related to surgeries in the occipital scalp region are conveyed via GON fibers. Blocking this nerve can be performed in a manner that is relatively quick, simple, and effective. Common surgical procedures in this region include the excision of benign and malignant tumors, scalp reduction surgery, and reconstructive procedures using free flaps, either following lesion excision or trauma [1,7].

This report details our center's experience with the application of GON block for analgesic purposes in three patients who underwent excision of malignant tumors followed by flap reconstruction in the occipital region of the scalp.

## Case presentation

This section provides the clinical details of the three cases reported herein. All patients provided written informed consent prior to participation in this case series. The characteristics of these patients are available in Table 1. All patients underwent balanced general anesthesia with the use of fentanyl, propofol, and rocuronium, and maintenance with sevoflurane. Following anesthetic induction and prior to surgical incision, a GON block was performed on the right or left side, depending on the laterality of the surgical site, using 4 mL of 0.375% ropivacaine. All blocks were performed by a specialized anesthesiologist.

**Table 1:** Characteristics of patients ASA: American Society of Anesthesiologists

Patient	ASA	Age	BMI (kg/m^2^)	Habits	Comorbidities
1	II	85	28.7	0	Hypertension; dyslipidemia
2	II	71	26.8	Smoker	Hypertension; type 2 diabetes mellitus
3	II	64	23.1	Smoker	Hypertension

With the patient in the prone position, ultrasound guidance was carried out with the use of a high-frequency linear transducer (13-6 MHz, Sonosite SII, FUJIFILM Sonosite, Inc., Bothell, WA, USA). Following skin antisepsis, the transducer was positioned over the external occipital protuberance and moved caudally to obtain a transverse view of the posterior arch of the atlas. The probe was then advanced further caudally to identify the C2, distinguished by its bifid spinous process. Once this key landmark was identified, the probe was moved laterally and along an oblique axis, with the medial end directed toward the C2 spinous process and the lateral end toward the transverse process of the atlas.

**Figure 1:**
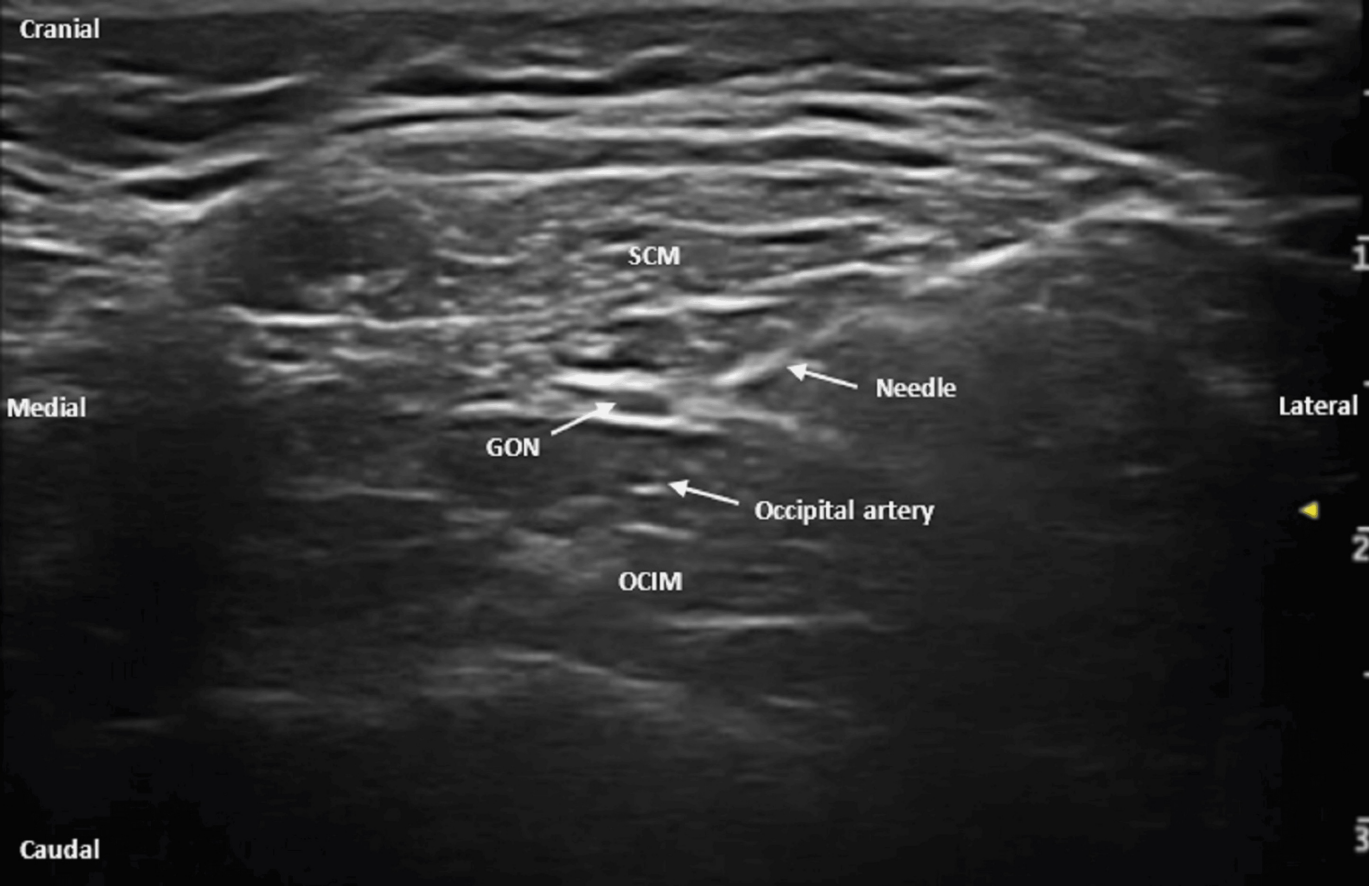
Ultrasound image of greater occipital nerve block SCM: semispinalis capitis muscles; GON: greater occipital nerve; OCIM: obliquus capitis inferior muscle

The obliquus capitis inferior muscle can be visualized in its entirety, extending from its insertion on C2 to the first cervical vertebra (C1), typically appearing hypoechoic in comparison to the semispinalis capitis muscle located superiorly. The GON was visualized between these two muscles and medial to the occipital artery, appearing as a hypoechoic, oval-shaped structure (Figure 1). A 25G, 50 mm needle was advanced in-plane in a lateral-to-medial direction, and the local anesthetic was administered.

During the intraoperative period, patients received additional intravenous analgesia, including 1 g of paracetamol and 2 g of metamizole. Postoperative analgesic prescriptions included paracetamol 1 g every eight hours, metamizole 1 g every eight hours, and tramadol 100 mg as needed (pro re nata (PRN)). Pain intensity was assessed using the numeric rating scale (NRS) immediately after surgery and at 2, 6, 12, and 24 hours following the nerve block. Additionally, the need for rescue analgesia was recorded.

Case 1

An 85-year-old patient, classified as American Society of Anesthesiologists (ASA) II, with a medical history of hypertension and dyslipidemia, was scheduled for excision of a basal cell carcinoma in the right occipital region with reconstruction using a bilobed flap. No clinically relevant changes were observed in the preoperative examinations. A right GON block was performed according to protocol, with no complications reported. In the immediate postoperative period, the patient reported zero pain, and throughout all subsequent assessments, pain remained ≤2, with no need for rescue analgesia.

Case 2

A 71-year-old patient, classified as ASA II, with a medical history of hypertension, type 2 diabetes mellitus, and smoking (45 pack-years), was scheduled for widening of surgical margins of a squamous cell carcinoma in the right occipital region, followed by closure using a local advancement flap. A preoperative evaluation was unremarkable. A right GON block was performed following the established protocol, with no complications. In the immediate postoperative period, the patient reported zero pain, and throughout the entire evaluation period, pain remained ≤1, with no need for rescue analgesia.

Case 3

A 64-year-old patient, classified as ASA II, with a medical history of hypertension and smoking (21 pack-years), was scheduled for excision of a squamous cell carcinoma in the left occipital region with reconstruction using a bilobed flap. Preoperative tests were within normal limits. A left GON block was performed in accordance with the protocol, and no complications occurred. In the immediate postoperative period, the patient reported zero pain, and during all subsequent assessments, pain remained ≤2, with no requirement for rescue analgesia.

Overall, all patients reported satisfactory pain relief during the first 24 hours following surgery (Table 2). Immediately after surgery, pain intensity measured by the NRS was 0 in all three patients. The median NRS scores at 2, 6, 12, and 24 hours were 0, 0, 1, and 2, respectively.

**Table 2:** Observed outcomes NRS: numeric rating scale

Patient	Postoperative pain assessment (NRS)				
	0 hours	2 hours	6 hours	12 hours	24 hours
1	0	0	0	1	2
2	0	0	0	1	1
3	0	0	0	0	2
Median	0	0	0	1	2

## Discussion

In this case series, we observed that GON block provided effective postoperative analgesia in patients undergoing excision of malignant tumors in the occipital region of the scalp, followed by reconstructive flap procedures. Pain intensity, assessed using the NRS, remained consistently low throughout the first 24 hours postoperatively, with a maximum median score of 2, which is considered mild pain (Table 2).

Postoperative pain is frequently reported by patients as one of the main complications following scalp tumor excision [8]. Our approach appears to have contributed to adequate postoperative pain control without the need for opioid administration. The only opioid use in these patients was limited to the induction phase of general anesthesia. These results are particularly relevant given the increasing emphasis on multimodal analgesia and enhanced recovery protocols in perioperative care.

A recent retrospective study analyzed opioid use in patients undergoing free flap reconstruction in the head and neck region [9]. The authors reported that 73% of patients received opioids at hospital discharge and more than half continued opioid use at their second postoperative follow-up [9]. Notably, among previously opioid-naïve patients, 20.3% were still using opioids up to four months after surgery [9]. These findings underscore the significant need for effective postoperative analgesia in surgeries involving the posterior scalp and support the potential role of the greater occipital nerve (GON) block as an opioid-sparing technique in this context.

In our center, these patients are typically prescribed 100 mg of tramadol as a rescue medication for pain control in the immediate postoperative period. Before we started using the GON block, we observed that the majority of these patients required two to three doses of tramadol within the first 24 hours postoperatively. This highlights the significant role that GON block can play in reducing reliance on traditional opioid-based pain management strategies, offering a more effective and potentially safer alternative.

The simplicity of the technique, particularly when guided by ultrasound, adds to its clinical appeal. A GON block can be performed in a manner that is relatively quick, simple, and effective. The most described side effects include erythema, localized edema, nerve or arterial injury, hematoma, and infection. When corticosteroids are used, localized alopecia at the injection site may also occur [10].

A few isolated reports, such as those by Finco et al. [3] and Mohammad et al. [4], have suggested that the GON block may reduce postoperative discomfort in scalp surgeries, but high-quality evidence remains scarce. Our case series contributes to this limited body of literature, providing further support for the feasibility and effectiveness of this technique in a surgical context.

## Conclusions

This case series suggests that GON block provided adequate analgesia, with pain intensity remaining mild and no need for rescue opioid analgesia. We believe that this case series provides a valuable contribution to the growing body of evidence supporting GON block as an effective method of postoperative pain control in scalp lesion excision surgeries involving the occipital region, enabling the use of an opioid-sparing analgesic strategy. Further studies with larger sample sizes and comparative designs are warranted to validate these findings and to establish standardized protocols for its use in this surgical context.
